# Dimensional psychotic experiences in adolescence: Evidence from a taxometric study of a community-based sample

**DOI:** 10.1016/j.psychres.2016.04.021

**Published:** 2016-07-30

**Authors:** Mark J. Taylor, Daniel Freeman, Angelica Ronald

**Affiliations:** a Centre for Brain & Cognitive Development, Department of Psychological Sciences, Birkbeck, University of London, UK; b Department of Psychiatry, University of Oxford, UK

**Keywords:** Psychosis, Taxon, Adolescence

## Abstract

Psychotic experiences of varying severity levels are common in adolescence. It is not known whether beyond a certain severity in the general population, psychotic experiences represent a categorically distinct phenomena to milder psychotic experiences. We employed taxometric analytic procedures to determine whether psychotic experiences in adolescence are taxonic (i.e. categorical) or dimensional. Six different psychotic experiences were assessed in a community sample of approximately 5000 adolescents. Three taxometric procedures were conducted. Across all procedures, there was no evidence of a taxon (i.e. a separate latent population) underlying psychotic experiences in adolescence. Rather, a dimensional structure was supported. The results support the notion that psychotic experiences are continuously distributed throughout the general population, and there is no clear discontinuity between milder and more severe psychotic experiences. Thus, these findings support the use of dimensional approaches to understanding psychotic experiences in etiological studies. In clinical practice, categorical cut-offs are needed: the present findings show that a ‘natural’ break point is not present for identifying severe psychotic experiences, and it is likely therefore that other criteria (such as general functioning) might better aid decision-making with regards to identifying individuals with severe psychotic experiences in need of care during adolescence.

## Highlights

• It is unclear if milder and severe psychotic experiences are categorically distinct.
• Taxometric analyses tested the structure of adolescent psychotic experiences.
• Adolescent psychotic experiences appeared to be dimensional, as opposed to taxonic.
• This study suggests no clear ‘break’ between mild and severe psychotic experiences.

## 1 Introduction

Psychotic experiences of varying severity levels, such as paranoia, hallucinations, and anhedonia, frequently occur in the general population (Laursen et al., 2007, Dominguez et al., 2011). They are common during adolescence, prior to the typical age of onset for a psychotic disorder (Poulton et al., 2000, Laursen et al., 2007, Ronald et al., 2014, Wong et al., 2014). Psychotic experiences confer increased risk of developing psychotic disorders (e.g. Kelleher and Cannon, 2011); they are also associated with elevated degrees of distress and risk of suicide (Brett et al., 2014, Kelleher et al., 2014), emphasizing the need to characterize and understand psychotic experiences in their own right.

An important question regards the structure of psychotic experiences in the general population; are they dimensional or categorical? If psychotic experiences are dimensional, then one would expect to see them quite commonly throughout the general population to varying degrees of severity. No clear cut between more severely affected and mildly affected individuals would be clear. To give an example, Binbay et al. (2012) investigated psychotic symptoms in a sample of Turkish adults. The association between these behaviors and proxies of genetic risk for psychosis (in this case, family history) was investigated. A greater family history of psychosis was associated with a linear increase in continuous psychotic symptoms, suggesting that milder and more severe forms of these symptoms present throughout the general population.

Conversely, it may be that psychotic experiences are categorical, with individuals with severe degrees of psychotic experiences presenting as qualitatively distinct from the remainder of the population. van Os et al. (2009) reported that psychotic experiences were indeed quite common throughout the general population. For the majority of individuals, however, these milder behaviors tended to disappear with development, while a minority of cases (approximately 7%) developed difficulties requiring clinical intervention, and were thus distinct from individuals showing milder degrees of psychotic experiences.

Evidence to date indicates that psychotic experiences are indeed present to varying degrees throughout the general population (Ronald et al., 2014). Further, a recent twin study reported that the heritability of psychotic experiences in adolescence was consistent across differing severity levels, suggesting that the genetic factors that influence severe degrees of psychotic experiences are also associated with milder degrees of psychotic experiences (Zavos et al., 2014), thus indicating that, on an etiological level, psychotic experiences are dimensional. A more recent development has been the recognition that individual psychotic experiences, such as paranoia (Freeman et al., 2005, Bebbington et al., 2013, Ronald et al., 2014) and lack of ability to experiences pleasure (Ronald et al., 2014), are also common in the general population, and present with varying degrees of severity.

Further direct evidence of continuous psychotic experiences in the general population could be gleaned from *taxometric procedures.* These methods were first proposed by Meehl (1962), and are designed to test for the existence of a *taxon* underlying a certain disorder. A taxon can be considered a latent population, who represent cases. The remainder of the population are considered to be controls (the *complement*). The taxon is qualitatively distinct from the complement. This is contrasted with the scenario whereby the disorder in question is dimensional; varying degrees of behaviors characteristic of a given disorder, which are qualitatively similar to those seen in individuals with the disorder, would be expected to present throughout the general population (Pickles and Angold, 2003). Meehl (1962) originally applied taxometric procedures to test for the existence of a schizotypy taxon. If such taxa exist, then the procedures enable one to estimate the base rate of the taxon (i.e. the prevalence rate).

Few studies have investigated whether psychotic experiences in the general population are continuously distributed. Psychotic experiences capture the symptoms characteristic of psychosis, such as paranoia, assessed as traits in the general population. In contrast, schizotypal traits comprise more personality-based scales such as magical ideation, that are less closely tied to the diagnostic criteria of psychotic disorders and conflate individual psychotic experiences. Two studies exist, and both lent support for the notion that psychotic experiences are dimensional. The first of these studies administered the Community Assessment of Psychic Experiences (CAPE) to a sample of college students; the evidence suggested that there was no taxon underlying psychotic experiences (Daneluzzo et al., 2009). Subsequently, a study of the Collaborative Psychiatric Epidemiological Survey and the National Comorbidity Survey, which focused solely on positive psychotic experiences in adults, also lent support for a dimensional, rather than taxonic, structure for psychotic experiences (Ahmed et al., 2012).

Notably, neither of these existing studies focused on psychotic experiences in adolescence. As mentioned above, adolescence is a time at which psychotic experiences are common, prior to the typical adult onset of psychotic disorders, and are risk factors for later psychotic disorders (Poulton et al., 2000, Laursen et al., 2007, Ronald et al., 2014), and hence there is a need to understand the structure of psychotic experiences in adolescence. For example, are psychotic experiences in adolescence continuously distributed, with no clear cut between more severely affected individuals and those showing milder manifestations? Or are psychotic experiences in adolescence taxonic, with adolescents showing more severe degrees of psychotic experiences presenting with qualitative differences to their peers? Such a question is of clinical and practical importance. For instance, if one is assessing psychotic experiences with a view to predict who will go on to develop a psychotic disorder, then it may help to understand whether established cut-offs are required, or whether one can rely on quantitative assessments of psychotic experiences. On a practical level, establishing whether psychotic experiences are taxonic may assist with etiological studies; should such studies rely on case-control approaches, which divide individuals into groups, or employ continuous trait scores?

To our knowledge, there are no studies employing taxometric procedures to characterize psychotic experiences in adolescence. We thus aimed to test whether adolescent psychotic experiences in a large, population-representative sample would represent a dimensional construct, or whether they would be underscored by a latent taxon. We focused on a community-based sample, as opposed to a clinically ascertained sample, to ensure that psychotic experiences would be present to differing degrees of severity, rather than biasing the sample towards more severe cases. Given existing evidence from alternative epidemiological methods (e.g. Binbay et al., 2012, Zavos et al., 2014), we expected psychotic experiences to be dimensional. We did not expect to find evidence of a latent taxon underlying psychotic experiences.

## 2 Method

### 2.1 Participants

Families taking part in the Twins Early Development Study (TEDS), a longitudinal sample of twins born in England and Wales between 1994 and 1996 (Haworth et al., 2013), were invited to take part in the Longitudinal Experiences And Perceptions (LEAP) project when the twins were aged 16-years. In total, 10,874 families were invited to participate in LEAP. 5059 parents (47%) and 5076 pairs of twins (47%) returned data; Table 1 shows various demographic characteristics of the participating and non-participating families. Participants were excluded from the study for genetic syndromes (including autism spectrum conditions, Fragile X syndrome, and cystic fibrosis), chromosomal abnormalities (including Down Syndrome and cerebral palsy), severe perinatal or prenatal complications, and missing first contact or zygosity data. Furthermore, in order to be included in the analyses, participants were required to have data available on all six psychotic experiences measures (detailed in the Materials section). Since twins do not constitute independent observations, one twin per pair was randomly selected for all analyses. The SPEQ scores of included twins, compared with non-included twins, are summarized in Supplementary Table 1. With the exception of anhedonia, included twins’ SPEQ scores did not significantly differ from those of non-included twins. The final sample thus comprised of 4721 participants, of whom 2614 were female and 2107 were male.

### 2.2 Measures

The Specific Psychotic Experiences Questionnaire (SPEQ; Ronald et al., 2014) is a multi-dimensional measure of psychotic experiences. The SPEQ comprised six subscales, constructed using principal components analysis (see Ronald et al. (2014)): Paranoia (15 items), Hallucinations (9 items), Cognitive Disorganisation (11 items), Grandiosity (8 items), Anhedonia (10 items), and Negative Symptoms (10 items). The items were all adapted from adult measures of psychotic experiences, with the wording modified for age appropriateness where necessary according to expert opinions from clinicians specializing in adolescent psychosis. All subscales were self-report, except for Negative Symptoms, which was completed by parents. For Paranoia and Hallucinations, the twins rated how frequently they had experienced the thoughts or experiences each item pertained to (e.g. ‘I need to be on my guard against others [Paranoia], ‘How often do you hear voices commenting on what you’re thinking or doing?’ [Hallucinations]). For the other three self-report subscales, participants were asked about these experiences during the previous month (‘Are you easily confused if too much happens at the same time?’ [Cognitive Disorganisation], ‘I have a special mission’ [Grandiosity], ‘I look forward to a lot of things in my life’ [Anhedonia]). For Negative Symptoms, parents were asked to rate their agreement with statements about the twins (e.g. ‘Often does not have much to say for him/herself’).

All six SPEQ subscales displayed good internal consistency (see Table 2), and were stable across a nine-month period (r=.65–.74). Construct validity was established using principal components analysis, reported elsewhere (Ronald et al., 2014), which supported the division of the SPEQ into six subscales. The SPEQ has been validated against a similar measure, the psychosis-like symptoms questionnaire (PLIKS; Zammit et al., 2011). Individuals who reported having ‘definitely’ experienced any psychotic symptoms on the PLIKS scored significantly higher on all SPEQ subscales, except for Anhedonia, than those who did not (all p<.001). Continuous scores on the PLIKS also displayed significant correlations (all p<.001) with Hallucinations (.60), Paranoia (.48), Cognitive Disorganisation (.41), and Grandiosity (.27) (Ronald et al., 2014). Finally, individuals with first- or second-degree relatives with schizophrenia and/or bipolar disorder scored higher on all SPEQ subscales (all p<.05, except for Hallucinations, which showed a trend in this direction), except Anhedonia. Density plots for the SPEQ subscales are given in Supplementary material.

### 2.3 Data analysis

Taxometric analyses were used to test whether psychotic experiences, as assessed by the SPEQ, are taxonic or dimensional. Significance of a finding is established through replication across multiple taxonic methods, as opposed to p-values (Waller and Meehl, 1998), hence three methods were used: mean above minus below a sliding cut (MAMBAC; Meehl and Yonce, 1994), maximum covariation (MAXCOV; Meehl and Yonce, 1995), and latent mode (L-MODE; Waller and Meehl, 1998). Before outlining these methods, the *indicators* of psychotic experiences used in these analyses are outlined.

#### 2.3.1 Indicators

Taxometric procedures assume that one has collected data on multiple indicators of the phenotype of interest. In this instance, the six SPEQ subscales were treated as indicators of psychotic experiences. Indicators are assumed to be valid predictors of the phenotype of interest. Validity information on the SPEQ is given in the Materials section. Furthermore, each indicator is assumed to represent a phenotypically distinct aspect of the disorder in question. The phenotypic distinction of the six SPEQ subscales was supported by an existing principal components analysis, which suggested that a six-factor solution best fit the SPEQ (Ronald et al., 2014).

In addition, the indicators included in analyses are assumed to correlate to an extent. Ruscio and Ruscio (2004) suggest that indicators should correlate at least .30 with one another in order to warrant inclusion in taxometric analyses. Table 2 shows the correlations between the SPEQ subscales. In light of these correlations, three indicators were selected for use in the analyses: Paranoia, Hallucinations, and Cognitive Disorganisation. All the analyses detailed below were conducted in R (R Core Team, 2013), using the syntax freely available from John Ruscio's website (http://www.tcnj.edu/~ruscio/taxometrics.html). The command line used in R, along with an explanation of each parameter, is given in Table 3.

#### 2.3.2 MAMBAC

This technique assumes that one has data on two indicators. One indicator is selected as the *input variable*. The sample is ordered on the basis of scores on the input variable, and is then cut into two groups on the basis of scores on the input variable, with successively increasing cut-offs used. A second indicator is selected as the *output variable*; mean differences in scores on the output variable are examined above and below the various cut-offs on the input variable. If a plot of the mean differences on the outcome variable against cut-offs on the input variable yields a curve with a clear peak, then this implies that there is a clear point at which the indicator distinguishes between high-scorers (a taxon) and lower scorers (a complement), and thus suggests a taxon underlying a trait. On the other hand, a flatter curve indicates that there is no clear cut between high- and low-scorers, instead supporting a dimensional construct. As we used three indicators in this study, MAMBAC was performed six times in a pairwise manner. The results were then averaged.

#### 2.3.3 MAXCOV

This method assumes that one has at least three indicators of a putative taxon. Covariance between two indicators is tested as a function of a third; that is, the sample is once again ordered on one indicator, then successively cut. Covariance between two other indicators is then tested above and below the cut. If a taxon exists, then plotting the covariances against the third indicator will yield a clear peak in the curve; such a curve suggests that covariance between two indicators differs as a function of the third, thus indicating the existence of two separable groups. If a trait is dimensional, however, the curve will be flatter, with no clear peak.

#### 2.3.4 L-MODE

All indicators are loaded onto a single factor in L-MODE, and factor scores are created for each participant. The factor scores are plotted; taxonic data will result in a bimodal distribution, whereby there is a group clearly displaying higher factor scores (the taxon) and a group displaying lower scores (the complement). Dimensional data, on the other hand, will result in a unimodal distribution; the factor scores do not support the existence of two separable groups within the sample.

#### 2.3.5 Other considerations

It is possible to generate taxonic and dimensional comparison datasets, which mimic the distribution and inter-indicator correlations present in the observed data (Ruscio, 2007). For each analysis, 100 taxonic and 100 categorical comparison datasets were generated. The results of these comparison datasets were then plotted against the observed results. There are two reasons for this; first, plotting the observed results against those expected if data are either taxonic or dimensional can assist with interpretation of the findings. Second, comparison datasets are useful for determining whether or not the observed data are able to adequately distinguish between taxonic and dimensional distributions (Ruscio et al., 2006, Ruscio, 2007). In generating comparison datasets, a fit statistic, comparison curve fit index (CCFI), can be calculated (Ruscio and Walters, 2009). CCFI falls between 0 and 1; the closer to 1 the estimate falls, the stronger the evidence of a taxon. Estimates closer to 0, however, support a dimensional construct. If, however, CCFI is between .4 and .6, then the data are unlikely to be adequate for distinguishing between taxonic and dimensional distributions (Ruscio and Walters, 2009). CCFI has previously been shown to be an accurate and sensitive mean of distinguishing between dimensional and categorical datasets (Ruscio et al., 2010).

It is possible to specify the expected base rate of a putative taxon prior to conducting taxometric analyses. To ensure that results were robust across procedures, analyses were performed twice: once with no taxonic base rate specified and once with a base rate of .07 (7%) specified, which was designed to mimic the proposed 7% prevalence of psychotic symptoms in adolescence (van Os et al., 2009, Kelleher et al., 2012).

Since skewed indicator variables can cause the tail end of MAMBAC and MAXCOV curves to rise, we applied log transformations to the SPEQ scales. Such scores were used in all analyses.

## 3 Results

The descriptive statistics for all six SPEQ subscales are shown in Table 1. The correlations between them are shown in Table 2.

### 3.1 MAMBAC

**Fig. 1:**
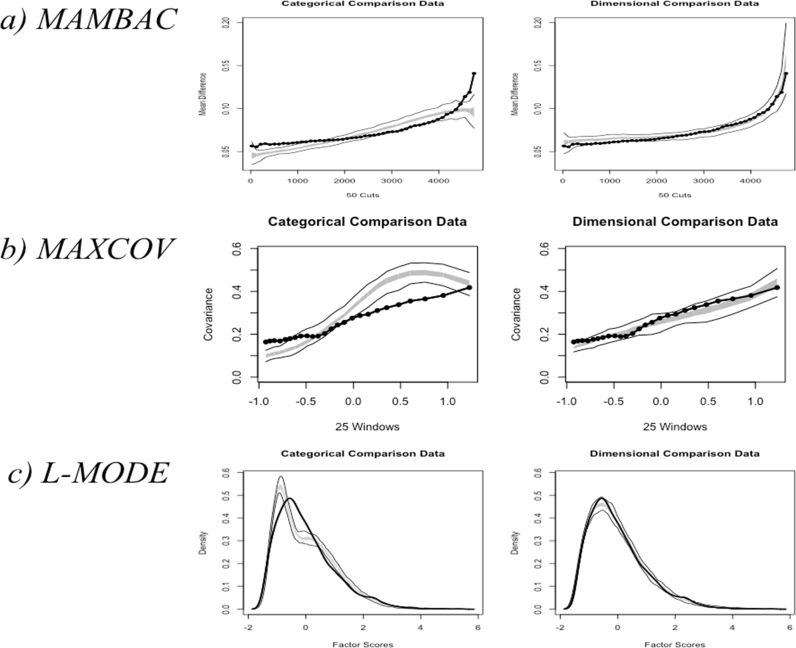
Graphs showing the results of taxometric analyses with no base rates specified. MAMBAC: mean above minus below a sliding cut; MAXCOV: maximum covariance; L-MODE: latent model. In Fig. 1(a), ‘cuts’ on the x-axis represent the positions in which the sample was cut, based on ordered input variable scores. The y-axis represent the mean difference in scores above and below each cut. In Fig. 1(b), ‘windows’ represent the cuts in the sample, with covariance between indicators plotted on the y-axis. In Fig. 1(c), factor scores are shown on the x-axis, with the density of each score plotted along the y-axis. The thick gray line represents the expected results for dimensional or categorical data for the middle 50% of the comparison datasets; the thinner gray lines either side of it represent the lower and upper bounds of the results. The result obtained from observed data are shown by the black lines.

The results of MAMBAC are shown in Fig. 1a. There are two graphs in the figure; in both, the solid black curve represents the averaged results for MAMBAC analyses of the observed data. The thick gray curve flanked by two thinner gray curves represents the curves that would be expected under dimensional or categorical models, based on comparison datasets. In examining these graphs, it is clear that the observed results were more consistent with a dimensional construct. Mean differences in scores on the output variables did not differ as a function of scores on the input variable, suggesting that the sample could not be divided into a taxon and a complement. Further, CCFI was estimated as .23, which is closer to 0 than 1, providing further support for a dimensional structure.

**Fig. 2:**
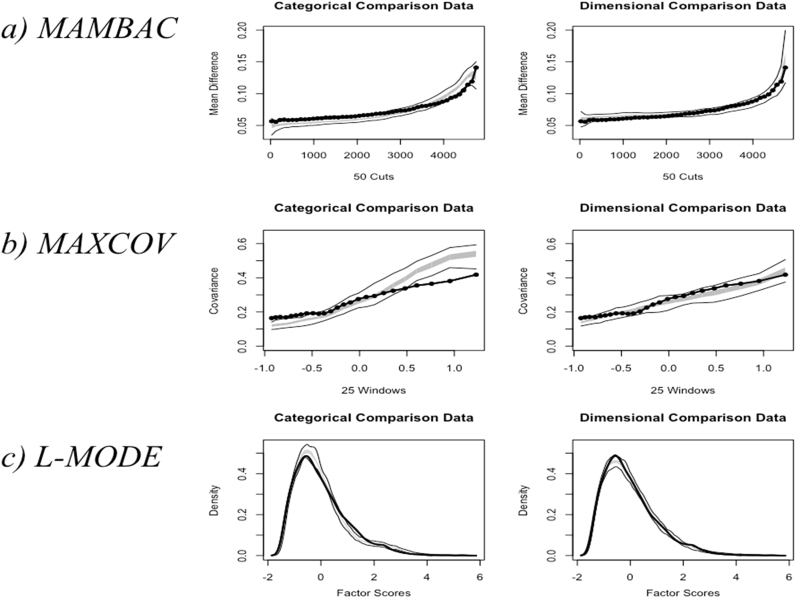
Graphs showing the results of taxometric analyses with a base rate of .07 specified. MAMBAC: mean above minus below a sliding cut; MAXCOV: maximum covariance; L-MODE: latent model. In Fig. 2(a), ‘cuts’ on the x-axis represent the positions in which the sample was cut, based on ordered input variable scores. The y-axis represent the mean difference in scores above and below each cut. In Fig. 2(b), ‘windows’ represent the cuts in the sample, with covariance between indicators plotted on the y-axis. In Fig. 2(c), factor scores are shown on the x-axis, with the density of each score plotted along the y-axis. The thick gray line represents the expected results for dimensional or categorical data for the middle 50% of the comparison datasets; the thinner gray lines either side of it represent the lower and upper bounds of the results. The result obtained from observed data are shown by the black lines.

Fig. 2a shows the equivalent results when a taxon base rate of .07 was specified. While the result was not as strong (CCFI=.26), CCFI was again closer to 0 than 1, and the shape of the curve for the observed results was closer to a dimensional distribution than a taxonic one. Indeed, there was no clear peak in the curve.

### 3.2 MAXCOV

Fig. 1b shows the MAXCOV results. The results are shown in the same manner as the MAMBAC results; the gray curves (including the thick curve and the two thinner curves either side of it) reflect the results from comparison datasets, while the black curve plots the observed results. These were more consistent with a dimensional construct. The curves were reasonably flat, showing no clear peak. CCFI was estimated as .14, again suggesting a dimensional structure for the observed data.

When a taxon base rate of .07 was specified, the results were still consistent with dimensional data. Fig. 2b presents graphs of the results. The observed results, as shown by the solid black curve, more closely matched the dimensional comparison data. CCFI was, again, closer to 0 than 1 (CCFI=.32).

### 3.3 L-MODE

Results for L-MODE are shown in Fig. 1c. Again, the solid black distributions show the results for the observed data (i.e. the frequency of the factor scores created from the three indicators). The gray lines on the left-hand graph show the result for taxonic comparison datasets, while the gray lines on the right-hand graph show the result for dimensional comparison datasets. The plot of the observed results showed a unimodal, rather than bimodal, distribution, consistent with dimensional data; CCFI was estimated as .19.

When a taxon base rate of .07 was specified, CCFI from L-MODE increased to .41. The distribution of factor scores was once again unimodal, supporting a dimensional structure.

Across the three taxometric procedures, results are strongly indicative that psychotic experiences are dimensional. With no base rate specified, the mean CCFI estimate across MAMBAC, MAXCOV, and L-MODE was .19. When a base rate of .07 was specified, the mean CCFI estimate was .33.

## 4 Discussion

To our knowledge, this is the first taxometric study of *adolescent* psychotic experiences, despite the potential utility of understanding if a taxon exists for severe psychotic experiences just prior to the age when psychotic disorders typically have their onset (Laursen et al., 2007). Using three different procedures, our findings provide no evidence of a taxon underlying psychotic experiences. Rather, a dimensional structure for psychotic experiences was supported. This is consistent with our expectations, and research using alternative methods (e.g. Binbay et al., 2012, Zavos et al., 2014), in showing no clear discontinuity between milder and more severe forms of psychotic experiences.

Diagnostic approaches to mental health problems have been anchored in the framework of the *Diagnostic and Statistic Manual* (DSM; APA, 2013); definitions of categories are required in order for clinicians to decide who requires treatment (Pickles and Angold, 2003). Further to this, such categories are potentially beneficial for creating health registries used in large-scale epidemiological studies, such as those that assess disorder prevalence and recurrence rates (e.g. Weiser et al., in press; Lundström et al., 2015). Nevertheless, for other purposes, such as basic research, it can be helpful to explore how symptoms behave in nature. In the case of psychotic experiences during adolescence, it is also the case that the serious psychiatric illnesses that they are phenotypically linked to, such as schizophrenia, have not typically begun. Taxometric analyses can test whether a separate taxon is present within manifestations of psychotic experiences in adolescence. The present findings show that a ‘natural’ break point is not present for identifying severe psychotic experiences in adolescence, and supports the current use of other criteria, such as general functioning, in decision-making by clinicians with regards to identifying individuals with severe psychotic experiences who are in need of care. Such an approach echoes recent calls to adopt more dimensional approaches to psychiatric disorders in research, such as the Research Domain Criteria (Sanislow et al., 2010).

It is important to stress that taxometric analyses cannot directly inform about the etiology of psychotic experiences, yet these findings still have important implications for designing genetic and environmental studies of psychotic experiences. Our findings indicate that such studies should strive to employ dimensional assessments of psychotic experiences. There are two key reasons for this. First, using dimensional assessments that account for all levels of severity allow for larger samples to be contacted, enhancing statistical power in etiological research (e.g. Zavos et al., 2014, Sieradzka et al., 2014). Second, imposing categories upon populations may also result in studies failing to appreciate the polygenic nature of psychotic experiences (Ruscio and Ruscio, 2008). For example, two studies recently investigated whether genes associated with clinical schizophrenia would also be linked with continuous measures of psychotic experiences in general population samples (Zammit et al., 2014; Sieradzka et al., 2014). Our findings offer empirical support for the use of dimensional assessments in etiological research on adolescent psychotic experiences.

As with any study, there are certain caveats to the findings presented here. The findings in this paper apply to positive and cognitive symptoms; while indicators included in a taxometric analysis need to be phenotypically distinct aspects of the construct in question, they also need to correlate to a certain degree. Negative psychotic experiences, for instance, did not correlate strongly enough with the other SPEQ subscales to warrant their inclusion in the analyses. Future work should aim to establish whether or not negative symptoms are also dimensional. This is particularly important given that studies of clinical samples suggest there may be a taxon underlying negative symptoms in individuals with schizophrenia (Blanchard et al., 2005, Ahmed et al., 2015). Thus, a pressing question will be to address whether adolescent negative symptoms in the community are dimensional or taxonic.

It is also important to note that the findings in this paper are evidence of dimensional psychotic experiences, but they do not suggest whether *individual* psychotic experiences follow a dimensional distribution. Recent studies are increasingly indicating that psychotic experiences do not comprise a single factor (e.g. Dominguez et al., 2011, Ronald et al., 2014), suggesting that the full picture is not as simple as a *psychosis continuum*, rather taxonic studies need to test for the existence of *psychosis continua*. We do not, however, have data on multiple indicators of each individual psychotic experience, such as paranoia and hallucinations, and so future studies need to further test the multi-dimensional nature of psychotic experiences using taxometric methods. Such studies could also employ complementary techniques, such as confirmatory factor analysis, to further delineate the exact nature of specific psychotic experiences. Additional confirmatory methods could be employed; for example, performing logistic regression analyses on various predictors of psychotic experiences, and ascertaining whether it is possible to assign group membership based on these predictors.

It should also be noted that there are multiple methods of selecting indicators for use in taxometric research. In the present study, we selected indicators based on correlations of greater than .30 (Ruscio and Ruscio, 2004). Alternative methods are available, however (e.g. Ruscio et al., 2011). These more contemporary approaches permit different criteria to be used in selecting taxon indicators. For instance, less stringent criteria of inter-indicator correlations of at least .21 could be used for a putative psychotic experiences taxon with an expected base rate of .07 (Ruscio et al., 2011). The taxometric analyses were conducted on a sample of twins, hence it is important to establish whether they extrapolate to singletons. Ronald et al. (2014) compared SPEQ scores in twins with those obtained from singletons, and found the mean scores did not differ significantly between twins and singletons. The two existing papers detailing taxometric analyses of psychotic experiences, which concurred with the results presented here, were based on singletons, yet did not focus on adolescent samples (Daneluzzo et al., 2009, Ahmed et al., 2012). It should be noted that the SPEQ measure was developed relatively recently and has not yet been used in multiple independent international samples. In addition, a confirmatory factor analysis on the SPEQ has not yet been conducted, which is a goal of future research. The SPEQ subscales also varied in the timeframe that the participants were asked to complete them for. Finally, there may be a small number of individuals with a diagnosis of a psychotic disorder in our sample, but this was not known in the sample.

To conclude, the taxometric analyses presented here suggest that, across three different analytic methods, there is no evidence of a latent psychotic experiences taxon in adolescence. Rather, psychotic experiences follow a dimensional pattern, whereby some individuals in the population will be expected to show milder degrees of psychotic experiences, while others will display considerably more, and may warrant clinical attention. The line between these individuals, however, is not clear, and so research that employs dimensional assessments of adolescent psychotic experiences is more allied to the underlying structure of these traits than research that imposes categorical definitions to divide individuals into separate severity groups.

## Contributors

MJT designed the study, conducted the statistical analyses, and prepared the manuscript. DF assisted in the design of the LEAP study and assisted in preparation of the manuscript. AR assisted in the design of this study and the LEAP study, held the grant that funded the LEAP study, and assisted in the preparation of this manuscript.

## Conflict of Interest

All authors have no competing interests to declare.

## Figures and Tables

**Table 1:** Descriptive statistics.

**Demographic characteristics**							
		Participating in LEAP			Non-Participating in LEAP		
% Male		45%			53%		
% Monozygotic		35%			32%		
% White		94%			91%		
% Mothers with one or more A-levels		16%			12%		
		
		
**Descriptive statistics**							
Measure	Possible range of scores	Interquartile range	$\bar{x}$ (SD)	Median		Skew	Cronbach's α
Paranoia	0–72	13	12.17 (10.62)	10		1.56 (−0.62)	.93
Hallucinations	0–45	7	4.66 (6.02)	2		2.09 (0.22)	.88
Cognitive Disorganisation	0–11	4	3.96 (2.85)	4		0.44 (−0.64)	.77
Grandiosity	0–24	6	5.32 (4.42)	4		1.18 (−0.41)	.86
Anhedonia	0–50	10	17.33 (7.93)	17		0.48 (−0.99)	.78
Negative Symptoms	0–30	4	2.81 (3.88)	1		2.42 (−0.85)	.86


*A-levels*: *qualifications taken at the age of 17*/*18 in England and Wales; LEAP*: *Longitudinal Experiences And Perceptions study*.

*Skew statistics in parentheses are for log transformed scores used in analyses*.

**Table 2:** Correlations between the SPEQ subscales.

	Paranoia	Hallucinations	Cognitive Disorgansation	Grandiosity	Anhedonia	Negative symptoms
Paranoia	–					
Hallucinations	.45**	–				
Cognitive Disorganisation	.41**	.40**	–			
Grandiosity	.09**	.20**	.01 (ns)	–		
Anhedonia	.08**	.02 (ns)	.03^1^	−.16**	–	
Negative Symptoms	.16**	.13**	.23**	−.01 (ns)	.14**	–


ns: non-significant.

** *p*<−.001.

* *p*<.05.

**Table 3:** R command lines.

**MAMBAC**
MAMBAC(ind,Comp.Data=T,N.Samples=100,Supplied.Class=F,Supplied.P=0,All.Pairs=T,N.Cuts=50)
ind: dataframe containing indicators
Comp.Data=T: generate comparison datasets
N.Samples=100: number of comparison datasets to generate
Supplied.Class=F: last column of dataframe does not contain categorical variable coding whether participants belong to taxon or complement
Supplied.P=0: specified base rate; 0 is used to indicate no base rate is specified. Changed to .07 when base rate of 7% was used
All.Pairs=T: use all possible pairings of indicators in analyses
N.Cuts=50: number of times to cut the sample into taxon and complement


**MAXCOV**
MAXEIG(ind,Comp.Data=T,N.Samples=100,Supplied.Class=F,Supplied.P=0,Windows=25,Calc.Cov=T)
ind: dataframe containing indicators
Comp.Data=T: generate comparison datasets
N.Samples=100: number of comparison datasets to generate
Supplied.Class=F: last column of dataframe does not contain categorical variable coding whether participants belong to taxon or complement
Supplied.P=0: specified base rate; 0 is used to indicate no base rate is specified. Changed to .07 when base rate of 7% was used
Windows=25: number of times to cut the sample into taxon and complement
Calc.Cov=T: command to calculate covariances, rather than Eigenvalues, and thus perform MAXCOV


**L-MODE**
LMode(ind,Comp.Data=T,N.Samples=100,Supplied.Class=F,Supplied.P=0)
ind: dataframe containing indicators
Comp.Data=T: generate comparison datasets
N.Samples=100: number of comparison datasets to generate
Supplied.Class=F: last column of dataframe does not contain categorical variable coding whether participants belong to taxon or complement
Supplied.P=0: specified base rate; 0 is used to indicate no base rate is specified. Changed to .07 when base rate of 7% was used.


*MAMBAC*: *mean above minus below a sliding cut*; *MAXCOV*: *maximum covariance*; *L-MODE*: *latent model*.

*Commands are based on the program authored by John Ruscio*, *freely available from his website* (http://www.tcnj.edu/~ruscio/TaxProg%202014-07-29. R).

## Footnotes

Appendix A Supplementary data associated with this article can be found in the online version at 10.1016/j.psychres.2016.04.021.

## References

[bib1] Ahmed A.O., Buckley P.F., Mabe P.A. (2012). Latent structure of psychotic experiences in the general population. Acta Psychiatr. Scand..

[bib2] Ahmed A.O., Strauss G.P., Buchann R.W., Kirkpatrick B., Carpenter W.T. (2015). Are negative symptoms dimensional or categorical? Detection and validation of deficit schizophrenia with taxometric and latent variable mixture models. Schizophr. Bull..

[bib4] American Psychiatric Association (2013). Diagnostic and Statistical Manual of Mental Disorders.

[bib5] Bebbington P.E., McBride O., Steel C., Kuipers E., Radovanovic M., Brugha T., Jenkins R., Meltzer H.I., Freeman D. (2013). The structure of paranoia in the general population. Br. J. Psychiatry.

[bib6] Binbay T., Drukker M., Elbi H., Tanik F.A., Özkinay F., Onay H., Zagli N., van Os J., Alptekin K. (2012). Testing the psychosis continuum: differential impact of genetic and nongenetic risk factors and comorbid psychopathology across the entire spectrum of psychosis. Schizophr. Bull..

[bib7] Blanchard J.J., Horan W.P., Collins L.M. (2005). Examining the latent structure of negative symptoms: is there a distinct subtype of negative symptom schizophrenia?. Schizophr. Res..

[bib8] Brett C., Heriot-Maitland C., McGuire P., Peters E. (2014). Predictors of distress associated with psychotic-like anomalous experiences in clinical and non-clinical populations. J. Clin. Psychol..

[bib9] Daneluzzo E., Stratta P., Di Tommaso S., Pacifico R., Riccardi I., Rossi A. (2009). Dimensional, non-taxonic latent structure of psychotic symptoms in a student sample. Soc. Psychiatry Psychiatr. Epidemiol..

[bib10] Dominguez M.D., Wichers M., Lieb R., Wittchen H.U., van Os J. (2011). Evidence that onset of clinical psychosis is an outcome of progressively more persistent subclinical psychotic experiences: an 8-year cohort study. Schizophr. Bull..

[bib11] Freeman D., Garety P.A., Bebbington P.E., Smith B., Rollinson R., Fowler D., Kuipers E., Ray K., Dunn G. (2005). Psychological investigation of the structure of paranoia in a non-clinical population. Br. J. Psychiatry.

[bib12] Haworth C.M., Davis O.S., Plomin R. (2013). Twins Early Development Study (TEDS): a genetically sensitive investigation of cognitive and behavioral development from childhood to young adulthood. Twin Res. Hum. Genet..

[bib13] Kelleher I., Cannon M. (2011). Psychotic-like experiences in the general population_ Characterizing a high-risk group for psychosis. Psychol. Med..

[bib14] Kelleher I., Connor D., Clarke M.C., Devlin N., Harley M., Cannon M. (2012). Prevalence of psychotic symptoms in children and adolescents: a systematic review and meta-analysis of population-based studies. Psychol. Med..

[bib15] Kelleher I., Cederlöf M., Lichtenstein P. (2014). Psychotic experiences as a predictor of the natural course of suicidal ideation: a Swedish cohort study. World Psychiatry.

[bib16] Laursen T.M., Munk-Olsen T., Nordentoft M., Bo Mortensen P. (2007). A comparison of selected risk factors for unipolar depressive disorder, bipolar affective disorder, schizoaffective disorder, and schizophrenia from a Danish population-based cohort. J. Clin. Psychiatry.

[bib17] Lundström S., Reichenberg A., Anckarsäter H., Lichtenstein P., Gillberg C. (2015). Autism phenotype versus registered diagnosis in Swedish children: prevalence trends over 10 years in general population sample. BMJ.

[bib18] Meehl P.E. (1962). Schizotaxia, schizotypy, schizophrenia. Am. Psychol..

[bib19] Meehl P.E., Yonce L.J. (1994). Taxometric analysis: I. Detecting taxonicity with two quantitative indicators using means above and below a sliding cut (MAMBAC procedure). Psychol. Rep..

[bib20] Meehl P.E., Yonce L.J. (1995). Taxometric analysis: II. Detecting taxonicity using covariance of two quantitative indicators in successive intervals of a third indicator (MAXCOV procedure). Psychol. Rep..

[bib21] Pickles A., Angold A. (2003). Natural categories or fundamental dimensions: on carving nature at the joints and the reariculation of psychopathology. Dev. Psychopathol..

[bib22] Poulton R., Caspi A., Moffitt T.E., Cannon M., Murray R., Harrington H. (2000). Children's self-reported psychotic symptoms and adult schizophreniform disorder: a 15-year longitudinal study. Arch. Gen. Psychiatry.

[bib23] R Core Team, 2013. R: A language and environment for statistical computing. R Foundation for Statistical Computing, Vienna

[bib24] Ronald A., Sieradzka D., Cardno A.G., Haworth C.M., McGuire P., Freeman D. (2014). Characterization of psychotic experiences in adolescence using the Specific Psychotic Experiences Questionnaire: findings from a study of 5000 16-year-olds. Schizophr. Bull..

[bib25] Ruscio J., Ruscio A.M. (2004). Clarifying boundary issues in psychopathology: the role of taxometrics in a comprehensive program of structural research. J. Abnorm. Psychol..

[bib26] Ruscio J., Haslam N., Ruscio A.M. (2006). Introduction to the Taxometric Method: a Practical Guide.

[bib27] Ruscio J. (2007). Taxometric analysis: an empirically-grounded approach to implementing the method. Crim. Justice Behav..

[bib28] Ruscio J., Ruscio A.M. (2008). Categories and dimensions advancing psychological science through the study of latent structure. Curr. Dir. Psychol. Sci..

[bib29] Ruscio J., Walters G.D. (2009). Using comparison data to differentiate categorical and dimensional data by examining factor score distributions: resolving the mode problem. Psychol. Assess..

[bib30] Ruscio J., Walters G.D., Marcus D.K., Kaczetow W. (2010). Comparing the relative fit of categorical and dimensional latent variable models using consistency tests. Psychol. Assess..

[bib31] Ruscio J., Ruscio A.M., Carney L.M. (2011). Performing taxometric analysis to distinguish categorical and dimensional variables. J. Exp. Psychopathol..

[bib32] Sanislow C.A., Pine D.S., Quinn K.J., Kozak M.J., Garvey M.A., Heinssen R.K., Wang P.S., Cuthbert B.N. (2010). Developing constructs for psychopathology research: research domain criteria. J. Abnorm. Psychol..

[bib33] Sieradzka D., Power R.A., Freeman D., Cardno A.G., McGuire P., Plomin R., Meaburn E.L., Dudbridge F., Ronald A. (2014). Are genetic risk factors for psychosis also associated with dimension-specific psychotic experiences in adolescence?. PLoS One.

[bib34] van Os J., Linscott R.J., Myin-Germeys I., Delespaul P., Krabbendam L. (2009). A systematic review and meta-analysis of the psychosis-continuum: evidence for a psychosis proneness-persistence-impairment model of psychotic disorder. Psychol. Med..

[bib35] Waller N.G., Meehl P.E. (1998). Multivariate Taxometric Procedures: Distinguishing Types from Continua.

[bib36] Weiser M., Kapara O., Werbeloff N., Goldberg S., Fenchel S., Reichenberg A., Yoffe R., Ginat K., Fruchter E., Davidson M. (2015). A population-based longitudinal study of suicide risk in male schizophrenia patients: proximity to hospital discharge and the moderating effect of premorbid IQ. Schizophr. Res..

[bib37] Wong K.K., Freeman D., Hughes C. (2014). Suspicious young minds: paranoid and mistrust in 8- to 14-year-olds in the U.K. and Hong Kong. Br. J. Psychiatry.

[bib38] Zammit S., Owen M.J., Evans J., Heron J., Lewis G. (2011). Cannabis, COMT and psychotic experiences. Br. J. Psychiatry.

[bib39] Zammit S., Hamshere M., Dwyer S., Georgiva L., Timpson N., Moskvina V., Richards A., Evans D.M., Lewis G., Jones P., Owen M.J., O’Donovan M.C. (2014). A population-based study of genetic variation and psychotic experiences in adolescents. Schizophr. Bull..

[bib40] Zavos H.M., Freeman D., Haworth C.M., McGuire P., Plomin R., Cardno A.G., Ronald A. (2014). Consistent etiology of severe, frequent psychotic experiences and milder, less severe manifestations: a twin study of specific psychotic experiences in adolescence. JAMA Psychiatry.

