# Supplementary material for: Dimensional psychotic experiences in adolescence: Evidence from a taxometric study of a community-based sample
Source: Psychiatry Res. 2016 Jul 30;241:35–42. doi: 10.1016/j.psychres.2016.04.021 (PMC4922386; doi:10.1016/j.psychres.2016.04.021)
Supplement: Supplementary file 1 — Supplementary material [file mmc1.docx]

**Taylor et al. Supplementary Materials**

***Table S1 R command lines***

| MAMBAC |
| --- |
| MAMBAC(ind,Comp.Data=T,N.Samples=100,Supplied.Class=F,Supplied.P=0,All.Pairs=T,N.Cuts=50) |
| ind: dataframe containing indicators  Comp.Data=T: generate comparison datasets  N.Samples=100: number of comparison datasets to generate  Supplied.Class=F: last column of dataframe does not contain categorical variable coding whether participants belong to taxon or complement  Supplied.P=0: specified base rate; 0 is used to indicate no base rate is specified. Changed to .07 when base rate of 7% was used  All.Pairs=T: use all possible pairings of indicators in analyses  N.Cuts=50: number of times to cut the sample into taxon and complement |
| MAXCOV |
| MAXEIG(ind,Comp.Data=T,N.Samples=100,Supplied.Class=F,Supplied.P=0,Windows=25,Calc.Cov=T) |
| ind: dataframe containing indicators  Comp.Data=T: generate comparison datasets  N.Samples=100: number of comparison datasets to generate  Supplied.Class=F: last column of dataframe does not contain categorical variable coding whether participants belong to taxon or complement  Supplied.P=0: specified base rate; 0 is used to indicate no base rate is specified. Changed to .07 when base rate of 7% was used  Windows=25: number of times to cut the sample into taxon and complement  Calc.Cov=T: command to calculate covariances, rather than Eigenvalues, and thus perform MAXCOV |
| L-MODE |
| LMode(ind,Comp.Data=T,N.Samples=100,Supplied.Class=F,Supplied.P=0) |
| ind: dataframe containing indicators  Comp.Data=T: generate comparison datasets  N.Samples=100: number of comparison datasets to generate  Supplied.Class=F: last column of dataframe does not contain categorical variable coding whether participants belong to taxon or complement  Supplied.P=0: specified base rate; 0 is used to indicate no base rate is specified. Changed to .07 when base rate of 7% was used. |

*MAMBAC: mean above minus below a sliding cut; MAXCOV: maximum covariance; L-MODE: latent model*

*Commands are based on the program authored by John Ruscio, freely available from his website (*[*http://www.tcnj.edu/~ruscio/TaxProg%202014-07-29.R*](http://www.tcnj.edu/~ruscio/TaxProg%202014-07-29.R)*)*

***Table S2 SPEQ scores for included and non-included twins***

| Scale | Included Twin $\bar{x}$ (SD) | Non-Included Twin $\bar{x}$ (SD) | t-test |
| --- | --- | --- | --- |
| Paranoia | 12.17 (10.62) | 11.96 (10.43) | t_4760_=0.96, p=.34 |
| Hallucinations | 4.66 (6.02) | 4.75 (6.17) | t_4770_=-1.08, p=.28 |
| Cognitive Disorganisation | 3.96 (2.85) | 3.97 (2.87) | t_4759_=0.62, p=.62 |
| Grandiosity | 5.32 (4.42) | 5.42 (4.55) | t_4739_=-1.45, p=.15 |
| Anhedonia | 17.33 (7.93) | 16.10 (7.75) | t_4742_=9.42, p<.001 |
| Negative Symptoms | 2.81 (3.88) | 2.82 (3.76) | t_4814_=-0.34, p=.73 |

*SPEQ: Specific Psychotic Experiences Questionnaire*

***Table S3 Density plots of the SPEQ subscales***
